# A Novel Transcriptional Regulator HbERF6 Regulates the HbCIPK2-Coordinated Pathway Conferring Salt Tolerance in Halophytic *Hordeum brevisubulatum*

**DOI:** 10.3389/fpls.2022.927253

**Published:** 2022-07-07

**Authors:** Ying Jiang, Haiwen Zhang, Yang Li, Congcong Chang, Yunxiao Wang, Hao Feng, Ruifen Li

**Affiliations:** ^1^Agro-Biotechnology Research Institute, Beijing Academy of Agriculture and Forestry Sciences, Beijing, China; ^2^Beijing Key Laboratory of Agricultural Genetic Resources and Biotechnology, Beijing, China; ^3^College of Life Science, Hebei Normal University, Shijiazhuang, China

**Keywords:** *Hordeum brevisubulatum*, salt tolerance, transcription factor, CIPK, halophyte

## Abstract

Halophytic *Hordeum brevisubulatum* is a perennial grass which has evolved many distinctive salt-adaptive mechanisms. Our previous studies indicated it could thrive under salt stress through maintaining better K^+^ and Na^+^ homeostasis. Stress-responsive HbCIPK2 can phosphorylate K^+^ channel HbVGKC1 and Na^+^ transporter HbSOS1L to prevent Na^+^ accumulation and K^+^ reduction, hence pathway was not detected in glycophytic plants. In this study, we cloned the inducible promoter of *HbCIPK2* by genome-walking, and identified a novel transcriptional regulator HbERF6 through yeast one-hybrid screening. HbERF6 functioned as a transcription factor which can bind to the GCC-box of the *HbCIPK2* promoter to activate its expression. *HbERF6* transgenic lines in *Arabidopsis* improved salt tolerance compared with wild type, and especially induced *AtCIPK24* (*SOS2*) expression, resulting in K^+^/Na^+^ homeostasis to enhance salt tolerance. All the results confirmed the inducible function of HbERF6 for CIPK genes during salt tolerance. This regulatory network that integrates transcriptional regulation and post-translation modification will unravel a novel salt stress-responsive mechanism, highlighting the value and utilization of the halophytic resource.

## Introduction

Soil salinization is a key environmental factor that adversely affects plant growth and crop productivity (Flowers and Yeo, 1995; Tester and Davenport, 2003). Better understanding of the plant salt tolerance mechanism benefits crop genetic improvement (Mansour et al., 2021). To adapt salt stress, plants develop a large number of physiological, biochemical, and molecular strategies to respond and defend to stress (Chen et al., 2018; Van Zelm et al., 2020). Till present, plenty of studies indicate that many genes are involved in salt stress response (Negrao et al., 2017). From salt sensing to signal transduction and downstream gene response, plants have evolved complex and hierarchical gene regulatory networks (Golldack et al., 2011). Each gene plays a crucial role in these regulatory networks, especially stress-responsive transcription factor networks in plants are attracting more and more attention (Song et al., 2016; Li et al., 2019).

Under stress, the concentration of Ca^2+^ in plant cytoplasm will change specifically with time and space. Calcineurin B-like protein (CBL) is a family of small proteins that can bind Ca^2+^ in plants. CBL and its target protein CBL-interacting protein kinase (CIPK) constitute the CBL-CIPK signal network system and play an important role in multiple abiotic and biotic stresses, such as drought, salt, low temperature, abscisic acid (ABA), nutrient deficiency, and pathogens. CBL-CIPK complexes control membrane transport through phosphorylating transporters and channels in the plasma membrane and tonoplast (Tang et al., 2020). The plasma membrane-localized AtCBL1/AtCBL9-AtCIPK23 complexes exhibit a synergistic effect of potassium and nitrogen nutrition in plants (Verma et al., 2021). AtCBL1/AtCBL9-AtCIPK23 modules phosphorylate the voltage-gated K^+^ channel AtAKT1 and promote plant K^+^ absorption under low-K^+^ condition. The same CBL-CIPK complexes regulate NO_3_^–^ and NH_4_^+^ uptake separately controlled by AtNRT1.1, a transporter for NO_3_,^–^ and an AMT1-type transporter. AtCIPK3, apple MdCIPK22, TaCIPK27, and so on regulate ABA response (Ma et al., 2017; Sanyal et al., 2017; Wang et al., 2018). AtCIPK26 and SlCIPK6 mediate ROS signaling pathways (Han et al., 2019; Ma et al., 2020). Although CIPKs of model plants and crops have been demonstrated to function in various stress responses, exact functions of other CIPKs in *Hordeum* lack more evidence, in addition to HbCIPK2 contributing to salt tolerance in wild barley (*Hordeum brevisubulatum*) (Li et al., 2012).

Salt response in higher plants occurs in two phases as follows: in the first phase, it responds to higher osmotic potential within an hour, while the second phase occurs mainly due to toxicity of sodium ions from long-term treatment of high salinity (Seifikalhor et al., 2019; Wu et al., 2021). Sodium ions impair potassium uptake and transport, thereby maintaining K^+^/Na^+^ homeostasis which is one of the elementary mechanisms for plants (Almeida et al., 2017; Zhang et al., 2020a). More obviously, K^+^ or Na^+^ balance is mainly controlled by respective transporters, whose activities are regulated by CBL-CIPK module-mediated pathways such as classical SOS and CBL-interacting protein kinase 23 (CIPK23) pathways (Qiu et al., 2002; Xu et al., 2006). CIPKs have been confirmed to be the hubs in many pathways and play important roles in plant stress regulatory networks (Ma et al., 2020; Tang et al., 2020). Moreover, some CIPK-encoding genes are responsive to various stresses, and their regulations in the transcriptional level still remain limited.

Transcription factors play a central role in plant response to different stresses, through binding to *cis*-acting elements in the promoters of target genes, or interacting with other transcription factors, which subsequently regulate gene expression (Singh et al., 2002; Wu et al., 2007). The ethylene responsive factor (ERF) is one of the unique transcription factor families, which features the conserved AP2/ERF domain in plants (Rehman and Mahmood, 2015; Xie et al., 2019). The ERFs function in plant stress response and the development process (Licausi et al., 2013; Feng et al., 2020). Previous reports have shown that several ERFs can be phosphorylated by MAPK cascade (Jung et al., 2010; Kishi-Kaboshi et al., 2010). However, it still remains unclear that the ERF transcriptional factor modulates the stress-responsive *CIPK* gene.

*Hordeum brevisubulatum* is a perennial halophyte in *Triticeae* referring to wild barley. It can grow well in saline-alkaline grassland in northern China as forage grass, and has evolved the salt tolerance mechanism during long-term adaption. Moreover, it is also wildly relative to cultivated barley and wheat, and therefore this wild species is a good resource for crop improvement of stress tolerance (Zhang et al., 2020b). However, less is known about its stress-responsive transcription factor networks. Our previous study indicated that this wild barley maintained K^+^/Na^+^ balance under salt stress, and stress-responsive HbCIPK2 conferred salt tolerance by preventing Na^+^ accumulation and K^+^ reduction (Li et al., 2012). Interestingly, a novel coordinated network was verified to link sodium and potassium regulatory pathways for HbCIPK2 as a hub (Zhang et al., 2020a).

In this study, we identified a novel transcriptional regulator of *HbCIPK2* and *HbERF6*, from wild barley through yeast one-hybrid (Y1H) library screening, confirming that HbERF6 can bind to the *cis*-regulatory element of the *HbCIPK2* promoter and activate the expression of *HbCIPK2*. Overexpression of *HbERF6* in *Arabidopsis* can improve the salt tolerance of the transgenic plants. Taken together, we discovered a positive upstream modulator of the *HbCIPK2*-mediated salt responsive network, playing an important role in salt stress.

## Materials and Methods

### Plant Growth Conditions

For *H. brevisubulatum* seedling growth, the procedures were adopted as described previously (Li et al., 2012). Wild-type (WT) and transgenic *Arabidopsis* plants were grown in a greenhouse (21–23°C, 16-h light/8-h dark with 60% relative humidity). For stress treatment, the 4-day-old seedlings grown on Murashige and Skoog media containing 0.5% sucrose and 0.8% agar (pH 5.7) were transferred to MS agar plates with 100 or 125 mM NaCl for another 8 days.

### *HbCIPK2* Promoter Cloning

The plant genomic DNA of *H. brevisubulatum* was extracted by the CTAB method, and the promoter of *HbCIPK2* was cloned by using the genome-walking kit (Takara). First, two reverse primers, SP1 and SP2, were designed according to the sequence of *HbCIPK2* CDS cloned in our laboratory. According to the operation instructions of the Takara kit, four kinds of restriction enzyme libraries were established with the wild barley genome as the template. The first phase of PCR amplification was performed with the non-specific primer AP1 provided by the kit and specific primer SP1. The second phase of nested amplification was performed with the first phase of PCR products as the template, and AP2 and SP2 as primers. After electrophoresis, the target bands were recovered and connected to the pEASY-Blunt cloning vector (TransGen Biotech) for sequencing. The basic *cis*-elements in the *HbCIPK2* promoter were analyzed using PlantCARE and PLACE online tools.

### Yeast One-Hybrid Library Screening

Total RNA was extracted from the shoots and roots of *H. brevisubulatum* seedlings, and subjected to 24 h salt-stress treatment (350 mM NaCl), using the TIANGEN RNA simple total RNA extraction kit. Y1H cDNA library was constructed using the Matchmaker™ Gold Yeast One-Hybrid System (Takara). The *HbCIPK2* promoter was ligated with the bait vector (pAbAi-pHbCIPK2). The bait vector was transferred into the yeast strain Y1HGold to prepare the competent cells, and the minimum inhibitory concentration of Aureobasidin A (AbA) was determined. The library plasmids were transferred into the bait strain which was cultured upside down at 30°C on SD/-Ura-Leu + AbA (300 mg/L) plates at 30°C for 3–5 days. A total of 126 positive yeast colonies were identified by PCR amplification with both T7 forward and AD reverse primers, respectively. After sequencing, the selected insert was amplified and cloned into the prey vector. The prey vector and the pAbAi-pHbCIPK2 vector were co-transformed into the yeast strain Y1HGold, and their growth was observed for further analysis. The full-length cDNA sequence of *HbERF6* was obtained from the positive prey vector mentioned above. Alignment of multiple sequences of the ERF family from different species was analyzed using the DNAMAN software, and the phylogenetic tree was constructed by the neighbor-joining method with the MEGA 7.0 software. The primers applied in this part are listed in Supplementary Table 3.

The interaction between the HbERF6 protein and *HbCIPK2* promoter was measured using the pairwise Y1H assay. The two GCC-boxes (box-1, −1086 to −1080; box-2, −232 to −226) of the *HbCIPK2* promoter were separately introduced into the pLacZi vector. The CDS sequence of the *HbERF6* gene was cloned and then inserted to the pB42AD vector. The two vectors of pB42AD-HbERF6 and pLacZi-GCC-box1/2 were co-transformed into yeast cells Y1HGold. The transformed yeast cells were grown on selective medium lacking Leu and Trp with X-gal at 30°C for 2–4 days. The primers applied in this part are listed in Supplementary Table 3.

### Quantitative Reverse Transcription PCR Analysis

The TRIzol reagent (Takara) was used to extract total RNA from the plant roots or shoots referring to Zhang’s paper (Zhang et al., 2020c). The purity (peak shape) and concentration of RNA were detected by using a spectrophotometer (NanoDrop™ 2000c). The sample cDNA was synthesized with the HiScript^®^ II Q Select RT SuperMix reagent Kit (Vazyme). The sample cDNA was diluted five times for real-time PCR (Bio-Rad, United States) with a SYBR mixture system (Vazyme). The gene expression analysis was performed using three biological and three technical replicates. The primers applied in this part are listed in Supplementary Table 3.

The effects of salt stress on plants include ion toxicity and osmotic stress. We selected five salt-stress-responsive genes that are related to osmotic adjustment and ion homeostasis, such as *AtCIPK24* (*At5G35410*), *AtCIPK2* (*At5G07070*), *AtCBL4* (*At5G24270*), *AtKIN2* (*At2G02800*), and *AtNHX1* (*At5G27150*). The ROS scavenging and salt-related enzyme-coding genes include *AtPOD* (*At3G49120*), *AtCAT1* (*At1G20630*), *AtP5CS* (*At3G55610*), and *AtADH* (*At1G77120*). In addition, we selected *AtCOR47* (*AT1G20440*) that accumulates in response to osmotic stress and two genes of *AtRD29B* (*At5G52300*) and *AtLEA3* (*At1G02820*) contributing to high salt response, respectively. The seedlings were grown on MS agar plates for 11 days, and then transferred to MS medium containing 100 mM NaCl for 10 days.

### Subcellular Localization

The *CaMV35:HbERF6-GFP* plasmid was constructed by amplifying the *HbERF6* CDS and ligating the sequence into the *Bam*HI and *Sal*I sites of the pCAMBIA1307 vector. After sequencing confirmation, *HbERF6-GFP* and *AtCBF1-RFP* (positive control) plasmids were co-transformed into the onion (*Allium cepa*) by micro-particle bombardments or barley protoplast by PEG-mediated transformation, and the plasmid containing only the *GFP* gene was also transformed as the negative control. After 24 h of dark culture at 25°C, the green fluorescent protein (GFP), red fluorescent protein (RFP), and DAPI fluorescence signals were observed using a laser confocal fluorescence microscope. The primers applied in this part are listed in Supplementary Table 3.

### GUS Stain Analysis

The promoter with a length of 1750 bp and the *GUS* gene was constructed into the pCAMBIA1381 vector, and then transformed into the WT *Arabidopsis* plants by the *Agrobacterium*-mediated floral-dip method. Notably, three T2 transgenic lines were selected, and five plants of each transgenic line were used for GUS staining and the process was repeated for three times. The 18-day-old transgenic *Arabidopsis* seedlings were grown on MS medium at 22°C and treated for 2 days with NaCl (100 mM), PEG (10%), and ABA (20 μM). The treated and untreated (control) seedlings were soaked in the GUS staining solution at 37°C in dark for overnight. The primers applied in this part are listed in Supplementary Table 3.

The *HbERF6* promoter (∼2 kb) was cloned using genome-walking mentioned above, and the vector pCAMBIA1381 with *pHbERF6:GUS* was constructed and introduced into *Agrobacterium tumefaciens* strain competent cells (GV3101). The genetic transformation of WT *Arabidopsis thaliana* was carried out through the floral dipping method. T2 transgenic lines were selected, and the 18-day-old transgenic *Arabidopsis* seedlings were grown on MS medium at 22°C and treated for 1 day with NaCl (100 mM). Then, the seedlings were immersed in GUS staining buffer, bleached with ethanol to remove the chlorophyll, and then photographed with a digital camera.

### *In vivo* Imaging and Firefly Luciferase Transient Assay

The promoter of *HbCIPK2* (up to ATG 1750 bp) was inserted to a *LUC* gene expression vector. The coding region of *HbERF6* was amplified and introduced into the pCAMBIA1307 vector as an effector plasmid. The reporter vector of *pHbCIPK2:LUC* and effector vector containing *HbERF6* gene were separately transformed into GV3101, and then the leaves of *Nicotiana benthamiana* were co-infiltrated. On the third day, after infection, each back blade was dropped in 300 μl of D-luciferin (Promega) with a final concentration of 1 mM, applied evenly, and then placed in dark for 2 min. The treated leaves were scanned and photographed with the plant living imaging system (Night SHADE LV 985), and the promoter activity was compared according to the fluorescence intensity. The primers applied in this part are listed in Supplementary Table 3.

### Purification of HbERF6 and Electrophoretic Mobility Shift Assays

The *HbERF6* coding fragment was amplified by PCR and inserted into a pET30a expression vector. The sequenced positive clones were used to transform BL21 (DE3) competent cells. The protein expression was examined by adding 0.5 mM isopropyl β-D-1-thiogalactopyranoside (IPTG) in transformed cells followed by incubation at 16°C for 20 h. Ni^2+^-NTA affinity columns (Cat R901-15, Invitrogen) were used to purify protein.

The GCC-box sequence in the *HbCIPK2* promoter was labeled by biotin at the 3′ end. The LightShift Chemiluminescent EMSA Kit (Thermo Fisher) was used to perform electrophoretic mobility shift assays (EMSAs). To each 20 μl reaction system containing 2 μl of 10× Binding buffer, 1 μl poly (dI.dC), and 2 μl biotin-labeled target DNA, 4 μg purified protein was added to the sample buffer at room temperature after 20 min of reaction. The protein-probe complex was all sampled, separated at 100 V for 40 min, and then transferred to the nylon membrane (380 mA, 40 min). The membrane was cross-linked under ultraviolet (120 mJ/cm^2^, 60 s) and then incubated with HRP labeled streptavidin. Finally, the images were exposed using the chemiluminescence imaging system.

### Dual-Luciferase Transient Assay

The *HbCIPK2* promoter sequence was inserted upstream of firefly luciferase (LUC) of the pGreenII 0800-LUC vector as a reporter gene. The CDS sequence of *HbERF6* was recombined into the pGreenII 62-SK vector as an effector gene. The negative control was an empty pGreenII 62-SK vector. *Agrobacterium* strain GV3101 carrying the reporter vector together with the effector vector strain was mixed with 1:1 suspension buffer and injected into the back of *N. benthamiana* leaves. The *N. benthamiana* plants were placed in a constant temperature incubator for 3 days. The firefly luciferase (LUC) and Renilla luciferase (REN) activities were measured using a chemiluminescence detector (Promega). Transcriptional regulation activity of HbERF6 on the *HbCIPK2* promoter was calculated using the LUC/REN ratio and normalized to the negative control.

### Genetic Transformation and Stress Treatment of *Arabidopsis thaliana*

*Agrobacterium* strain GV3101 with the *CaMV35:HbERF6* construct was used to infect *A. thaliana* (Ecotype Colombia-0) using the floral-dip method. Notably, three lines (L6, L7, and L13) of *Arabidopsis* T3 transgenic plants were selected to assess salt tolerance. For identification of salt stress tolerance in the transgenic seedlings, the seedlings cultured on MS medium for 4 days were transferred to MS medium supplemented with 100 and 125 mM NaCl for another 8 days. Then, the fresh weight and root length of the seedlings were measured and analyzed. Transgenic lines and WT were seeded in soil to observe salt stress tolerance. When the seedlings were grown at the 6th leaf stage, they were treated with 350 mM NaCl solution once in every 7 days for two times. After normal watering and culturing for 14 days, the survival rates of plants were investigated and photographed.

### Sodium and Potassium Ion Content Measurements

The 11-day-old seedlings grown on MS medium were transferred to MS medium plates containing l00 mM NaCl for 10 days. The plants were digested by acid solution and the concentrations of K^+^ and Na^+^ were analyzed using an inductively coupled plasma atomic emission spectrometer (ICP-AES) according to the methods in the previous study (Li et al., 2012).

### Determination of Total Chlorophyll Content

Fresh plant leaves (approximately 0.1 g) were fully ground under dark or low light conditions. According to the description of the plant chlorophyll content detection kit (Solarbio, BC0990), the extract solution (100% ethanol:acetone = 1:2) was added to the grated tissue and reacted for about 3 h, until the color of the tissue residue was close to white. The absorbance was measured with an ultraviolet spectrophotometer (UV-1800PC) at the wavelengths of 645 and 663 nm, respectively. The total chlorophyll content was calculated referring to the kit method.

### Data Processing and Analysis

The abovementioned procedures were repeated at least three times. We used the SigmaPlot software for statistical data processing. The data were statistically analyzed using one-way ANOVA (*P < 0.05, ^**^P < 0.01, ^***^P < 0.001). The results are expressed as mean ± SD.

## Results

### Discovery and Analysis of the *HbCIPK2* Promoter

**FIGURE 1:**
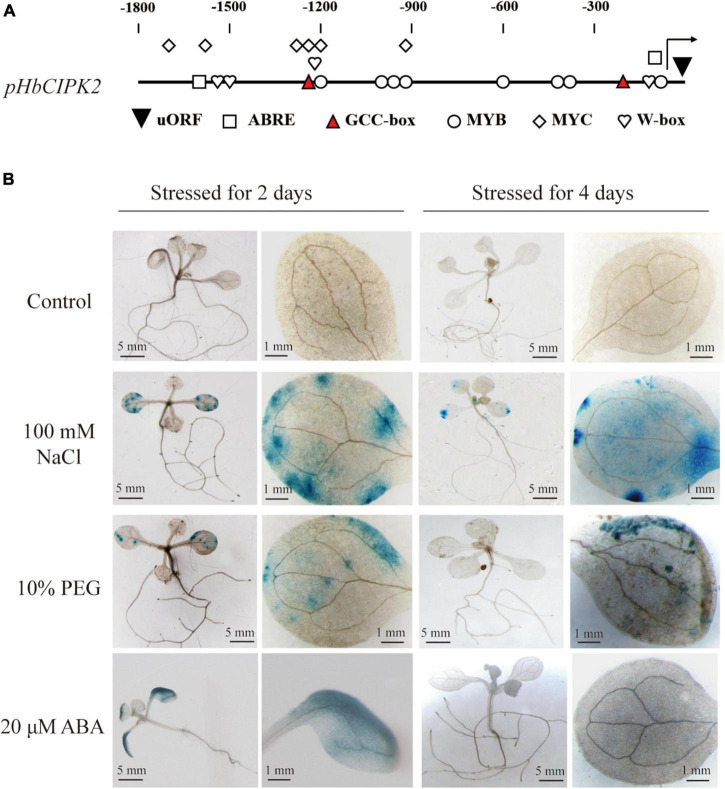
The activity of *HbCIPK2* promoter was induced under different stresses. **(A)** Structural diagram of *HbCIPK2* promoter, *HbCIPK2* promoter contains two GCC-boxes (box-1, –1086 to –1080; box-2, –232 to –226). Different components are represented by different icons. GCC boxes are denoted *via* triangles. **(B)** Histochemical staining of transgenic *Arabidopsis* lines under control and various stress conditions at different times. Scale bars are marked in the figures.

Previously, we reported *HbCIPK2* from halophytic *H. brevisubulatum* conferred salt tolerance which was inducible by abiotic stress, which indicates that *HbCIPK2* may be regulated by one type of transcriptional factor. However, the genome-wide sequences of *H. brevisubulatum* are unknown, until then we had to apply the genome-walking procedure to clone the promoter sequence of *HbCIPK2*. Finally, the fragment with a length of 1750 bp was obtained and identified as the target sequence. There are two GCC-box elements (AGCCGCC) at positions −1086 to −1080 and −232 to −226 in the *HbCIPK2* promoter sequence, respectively. A large number of basic *cis*-elements such as CAAT and TATA boxes were found in the promoter using the PlantCARE and PLACE online tools (Supplementary Table 1). In addition, the promoter of *HbCIPK2* also contains several *cis*-acting elements related to abiotic stress, such as salt response element, hormone response element, and dehydration response element (Figure 1A). The promoter sequence further suggested that *HbCIPK2* might respond to abiotic stress, and it will provide the possibility for the discovery of the *HbCIPK2* regulatory factor.

To analyze the activity of the *HbCIPK2* promoter, the fusion expression vector of *pHbCIPK2:GUS* was constructed. The transgenic plants were screened for T2 generation and subjected to different stress treatments. GUS staining was almost not observed without treatment, but increased after 10% PEG or 20 μM ABA stress treatment. The transgenic seedlings exhibited maximum GUS staining after 100 mM NaCl treatment (Figure 1B). These results showed that the *HbCIPK2* promoter has inducible activities under drought, ABA, and salt stress.

### Identification of the Transcription Factor *HbERF6* Through Yeast One-Hybrid Screening

**FIGURE 2:**
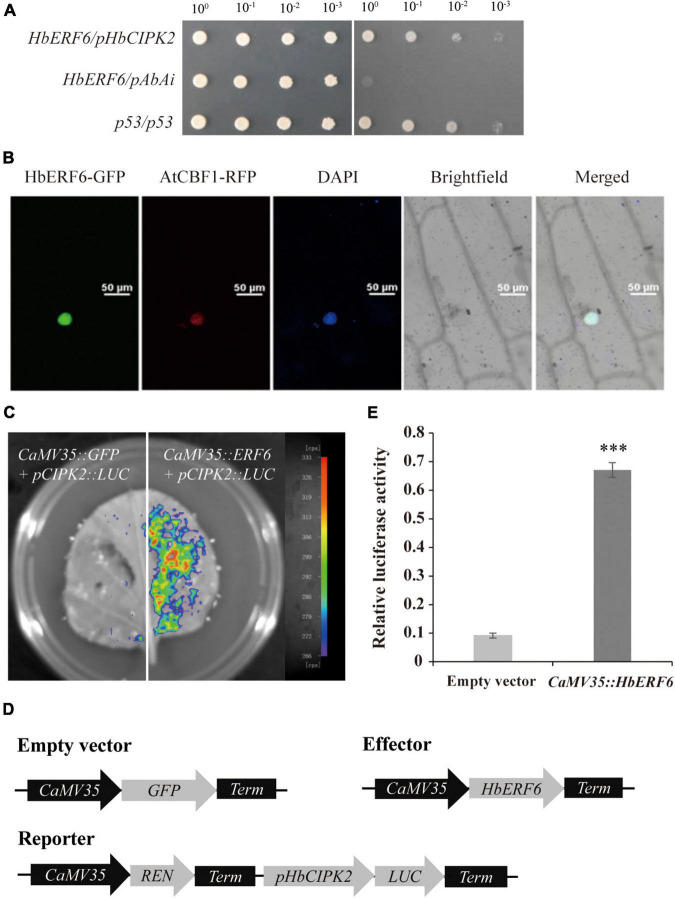
HbERF6 can interact with the *HbCIPK2* promoter in yeast and activate *HbCIPK2* in *N. benthamiana* leaves. **(A)** Interaction of the HbERF6 and *HbCIPK2* promoter was determined based on the ability of transformed yeast to grow on SD/-leu and SD/-Leu/AbA (300 mg/L). **(B)** Nuclear localization of the HbERF6-GFP fusion protein in onion cells. Bar = 50 μm. **(C)** A luciferase imaging assay indicated that HbERF6 can activate the expression of *HbCIPK2*. **(D)** Diagrams of relevant vectors used in the dual luciferase assay. *HbERF6* was cloned into the pGreenII 62-SK vector as an effector, the promoter of *HbCIPK2* was inserted into the pGreenII 0800-LUC vector as a reporter, and each of REN, Renilla luciferase, was used as an internal control. **(E)** The relative LUC activities indicated that HbERF6 can enhance the promoter activity of *HbCIPK2* in *N. benthamiana* leaves. Statistical analysis was performed by one-way ANOVA; asterisks indicate significant variation (^***^P < 0.001).

To screen the regulatory factor for *HbCIPK2*, we used the Y1H system and the strategy of the full-length promoter due to uncertain *cis*-element. The 1750 bp promoter of *HbCIPK2* was used as bait to screen the cDNA library from the plants subjected to salt stress (Figure 1A). Interestingly, among 126 colonies growing and conferring resistance to higher AbA (Supplementary Figure 1A), after PCR amplification, 65 positive yeast colonies with longer than 800-bp fragments were selected to sequence. After sequencing and blast analysis, two prey clones were confirmed to be the homologous gene of transcription factor *ERF6*, which was named as HbERF6. The prey plasmid with *HbERF6* and the bait plasmid with the promoter of *HbCIPK2* were co-transformed into the yeast Y1HGold strain that could grow on the SD/-leu-ura medium containing AbA (Figure 2A). This indicated that HbERF6 might be a regulatory factor of the *HbCIPK2* gene.

*HbERF6* (GenBank No. MZ935744) contains a 1029 bp intact open reading frame, encoding a polypeptide with 343 amino acids residues. The deduced N-terminus of HbERF6 contains some acidic amino acids as an activation domain, and the C-terminus enriches arginine (R) and lysine (L), showing a nuclear localization signal (Supplementary Figure 1B). The HbERF6 protein contains only one conserved AP2/ERF domain as a key element binding to the target DNA, and the 14th and 19th amino acids of which are alanine (A) and aspartic acid (D), respectively, suggesting that it belongs to the ERF family (Supplementary Figures 1B,D). The tertiary structure of the HbERF6 AP2/ERF domain (151-213aa) was predicted by online software SWISSMODEL. The AP2/ERF domain contains three β-sheets (β-1-β-3) in the N-terminal and 1 α-helix (α) in the C-terminal (Supplementary Figure 1C). The β-3 sheet and α-helix constitute a conserved RAYD element. β-1 and β-2 form a YRG element rich in hydrophilic amino acids (Supplementary Figure 1E). The sequenced PCR results using the genomic DNA as the template showed that the *HbERF6* gene has no intron. The analysis of the phylogenetic tree indicated that the HbERF6 protein shows higher homology with HvERF5 (GenBank No. ANA52685), AtERF5 (AT5G47230), ZmERF5 (GenBank No. PWZ220596.1), and OsERF5 (GenBank No. XP015624058), respectively (Supplementary Figure 1E).

### HbERF6 Can Activate *HbCIPK2*

To identify the regulated function of HbERF6, first, we identified the subcellular localization of HbERF6. The expression vector of HbERF6 fused with GFP was constructed, and the marker protein AtCBF1 fused with RFP acted as a positive control. The construct of *HbERF6-GFP* derived by the *CaMV35* promoter was bombarded into onion epidermal cells. By using confocal microscopy, HbERF6-GFP fusion protein in cells was predominantly co-located at the nucleus, and coincided with marker protein AtCBF1 and DAPI staining (Figure 2B), as GFP protein without fusion HbERF6 expressed in the whole cells (Supplementary Figure 2).

We next performed a firefly luciferase (LUC) imaging assay to demonstrate that HbERF6 activated the expression of *HbCIPK2 in vivo*. Constructs harboring *LUC* under the control of the *HbCIPK2* promoter and *CaMV35:HbERF6* were co-infiltrated into *N. benthamiana* leaves to transiently detect the LUC activity. We can detect the strong LUC activity in *N. benthamiana* leaves, but no LUC activity was observed in the negative control (Figure 2C). Furthermore, we used the effector-reporter system to analyze the LUC transcriptional activity (Figure 2D). The LUC activity driven by the promoter of *HbCIPK2* was obviously upregulated in transiently overexpressing *HbERF6 N. benthamiana* leaves, indicating that HbERF6 interacted with the promoter of *HbCIPK2* and positively regulated the expression level of *HbCIPK2* (Figure 2E). These results showed that *HbCIPK2* is the downstream target of *HbERF6*.

### HbERF6 Binds to the GCC *Cis*-Element

**FIGURE 3:**
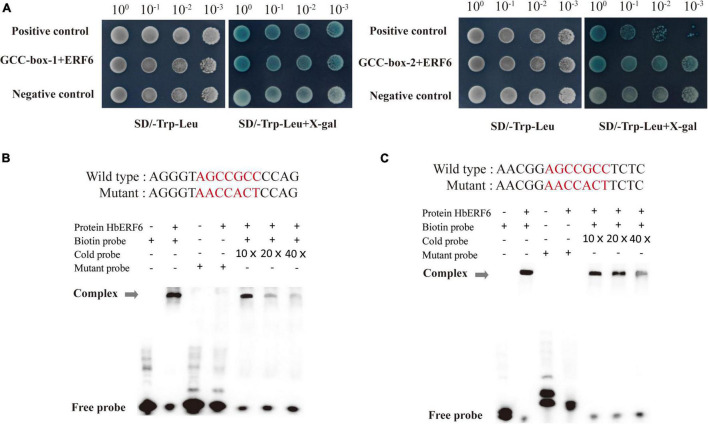
HbERF6 directly binds to GCC-boxes of the *HbCIPK2* promoter. **(A)** Yeast one-hybrid assay showed that HbERF6 binds to the GCC-boxes in the promoter of *HbCIPK2* (GCC-box-1 and GCC-box-2). The transformants were screened by SD/-Trp-Leu and SD/-Trp-Leu + X-gal for 3 days. Electrophoretic mobility shift assays (EMSAs) showed that HbERF6 can, respectively, bind to GCC-box 1 **(B)** and 2 **(C)** of the *HbCIPK2* promoter. A mutant labeled probe with AACCACT to replace the GCC-box (AGCCGCC) was used as a negative control. 10×, 20×, and 40×, respectively, represent the ratio of amounts of non-biotin-labeled probes to biotin-labeled probes. Gray arrows indicate the positions of DNA-protein complexes.

To confirm the binding sites of HbERF6 to the promoter of *HbCIPK2*, we analyzed the sequence of the promoter, and found two GCC-boxes (*pHbCIPK2*GCC-box-1, −2) within 1750 bp before the ATG start codon. The GCC-boxes (sequence: AGCCGCC) were located at positions −230 and −1086, respectively (Figure 1A). The two 16-bp repeat fragments of the *HbCIPK2* promoter containing the GCC-box were transformed into yeast cells, respectively, accompanying by *HbERF6*. The result showed that HbERF6 could bind to the GCC-box of the *HbCIPK2* promoter using the Y1H system (Figure 3A). Furthermore, we designed two pairs of probes targeting each one of the GCC-boxes according to the 16 bp sequence of Y1H assays. We carried out EMSA experiments to examine the interaction between HbERF6 and the *HbCIPK2* promoter. The recombinant protein His-HbERF6 directly could bind both of the GCC-boxes of *HbCIPK2* promoter *in vitro*, but the mutant probe had no combination with HbERF6 (Figures 3B,C). Therefore, all the results confirmed the recognition and interaction between HbERF6 and the GCC-box of the *HbCIPK2* promoter.

### *HbERF6* Is a Stress-Responsive Gene

**FIGURE 4:**
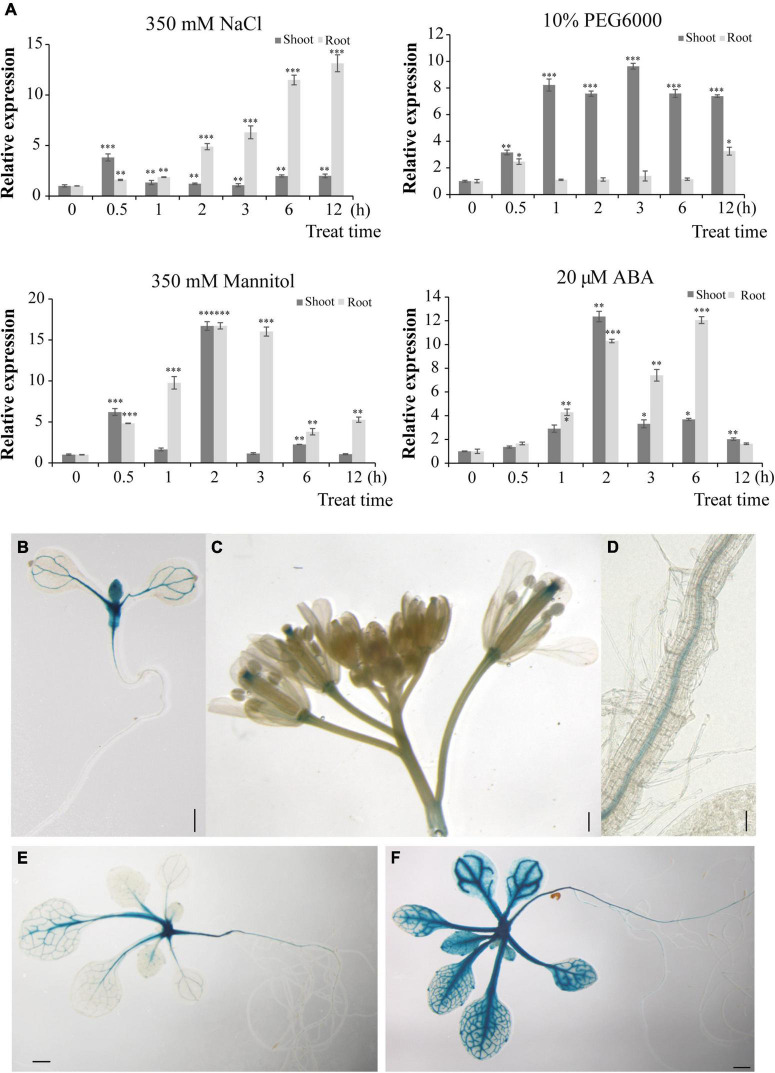
*HbERF6* is inducible under different stress conditions and GUS activity is mediated by the *HbERF6* promoter in transgenic *Arabidopsis* plants. **(A)** Transcript levels of *HbERF6* in roots and shoots of wild barley at two-leaf and one-heart stage suffering from salt (350 mM NaCl), water deficiency (10% PEG6000 and 350 mM mannitol), and ABA (20 μM) using qRT-PCR. *18SrDNA* was used as an internal reference gene. Each value is the average of three biological replicates and error bars mean ± SD. Asterisks indicate significant variation (*P < 0.05, *P < 0.01 and ^***^P < 0.001). **(B)** The 8-day-old *pHbERF6:GUS* seedlings after imbibition of seeds, **(C)** inflorescence, and **(D)** primary roots, the 15-day-old seedlings after imbibition of seeds in MS medium **(E)** and after 100 mM NaCl treatment for 16 h **(F)**. Scale bars = 2 mm in **(B)**, 1 mm in **(C–E)**, and 400 μm in **(F)**.

To identify the initial response of *HbERF6* to different stresses in *H. brevisubulatum*, we detected the expression of *HbERF6* in shoots and roots during the early stage of salt, drought, and ABA stress. We treated the 3-week-old plants of *H. brevisubulatum* under 350 mM NaCl stress for time series, and then the RNAs of shoots and roots were separately extracted. Using real-time PCR, we found that the transcripts of *HbERF6* were significantly inducible in roots than in shoots under salt treatment. In contrast, *HbERF6* in shoots strongly responded to 10% PEG6000 in shoots than in roots. It showed a similar expression pattern under 350 mM mannitol and 20 μM ABA (Figure 4A). In general, *HbERF6* was obviously upregulated under salt treatment, especially in the roots, which was coincident with the reported physiological function of HbCIPK2 (Li et al., 2012).

To establish the tissue-specific expression of HbERF6, transgenic plants in *Arabidopsis* expressing the *GUS* reporter gene driven by the *HbERF6* promoter (∼2 kb upstream from ATG) were detected. Notably, 8 days after imbibition, the strong GUS expression was observed in the shoot apex zone and the vascular bundle of cotyledons (Figure 4B). At the reproductive stage, GUS activity was detected mainly in the style and pedicles of inflorescences, and also expressed in the pollen tube of the gynoecium and the stamen filaments (Figure 4C). *GUS* expression was also detected in the vasculature of primary roots (Figure 4D). Notably, the 15-day-old seedlings showed weak GUS activity in the vascular tissues of the leaves and primary roots as well as in the shoot apex in MS medium (Figure 4E). However, when the plants were treated with 100 mM NaCl for 1 day, stronger GUS activity was observed in the vascular tissues of the leaves as well as in the shoot apex, and it was examined in the primary and lateral roots (Figure 4F). Obviously, *pHbERF6:GUS* activity of the transformed plants was strongly induced under salt stress.

### Overexpression of *HbERF6* Improves Salt Tolerance of *Arabidopsis*

**FIGURE 5:**
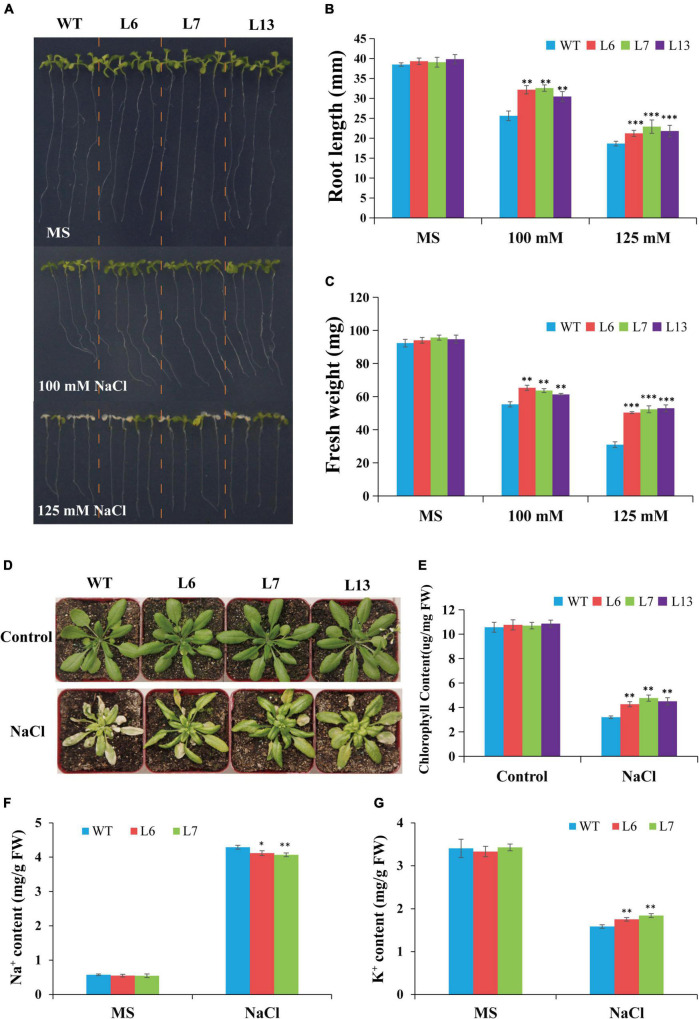
Comparison of salt tolerance between wild type (WT) and transgenic lines. **(A)** Growth and development of the *HbERF6*-overexpressing lines and WT seedlings under NaCl treatment and normal condition. **(B,C)** Root length and fresh weight of the 12-day-old seedlings on MS plates with and without salt were measured, respectively. **(D)** Representative photographs of potted plants of WT and overexpressing *HbERF6* plants at 6th leaf stage which have been treated with 350 mM NaCl solution once in every 7 days for two times. After that, watering and culturing were performed for about 14 days. No treatment was set as control. **(E)** The chlorophyll content of overexpressing lines and WT was measured just after recording the phenotype in **(D)**. The Na^+^ **(F)** and K^+^ **(G)** contents of the 11-day-old transgenic and WT seedlings were measured under normal condition and salt stress (100 mM NaCl for 10 days). The experiments were repeated three times. Asterisks show that the difference in the values is significant between transgenic lines and the WT (*P < 0.05; ^**^P < 0.01; ^***^P < 0.001).

To determine the role of *HbERF6* in plants, transgenic *A. thaliana* lines overexpressing *HbERF6* were analyzed. We compared the growth and development between the *HbERF6* overexpressing lines (L6, L7, and L13) and WT plants subjected to salt stress. Under normal condition (control), the transgenic *Arabidopsis* seedlings had no difference with WT; however, the transgenic lines showed longer roots and larger leaves than WT on MS medium containing 100 mM NaCl for 8 days. The WT plants displayed more serious wilting than the transgenic lines following the 125 mM NaCl treatment for 8 days (Figure 5A). The fresh weights and root growth rates were significantly greater in the *HbERF6*-overexpressing lines than those in the WT plants treated with 100 or 125 mM NaCl (Figures 5B,C).

To further confirm the *HbERF6* function in response to salt tolerance, the phenotype of the transgenic *Arabidopsis* and WT plants in the adult stage was observed. The transgenic lines overexpressing *HbERF6* and WT plants were cultivated until the 6th leaf stage in the greenhouse, and then they were watered with 350 mM NaCl solution once in every 7 days for two times. After treatment, they recovered for 14 days under normal condition for recording their phenotype. The *HbERF6*-overexpressing lines showed better growth than the WT plants during the adult stage (Figure 5D). The chlorophyll content of transgenic plants was significantly higher than that of the WT plants (Figure 5E). These results indicated that the transgenic plants overexpressing *HbERF6* exhibit enhanced salt tolerance.

To demonstrate the different effect of salt stress on the accumulation of Na^+^ and absorption of K^+^, the ion content of the 11-day-old transgenic and WT seedlings was examined under normal condition (0 mM NaCl) and salt stress (100 mM NaCl for 10 days) with ICP-AES. For Na^+^ or K^+^ content, no significant differences were detected between transgenic and WT plants under normal condition. However, the increase of Na^+^ in transgenic *Arabidopsis* was significantly lower than that of WT under salt treatment (Figure 5F). The decrease in K^+^ of WT plants was significantly higher than that of transgenic seedlings under the same treatment (Figure 5G), suggesting that *HbERF6* overexpressing can prevent K^+^ reduction and Na^+^ accumulation to reach K^+^/Na^+^ homeostasis in *Arabidopsis*.

### HbERF6 Activates the Expression of Stress-Responsive Genes

**FIGURE 6:**
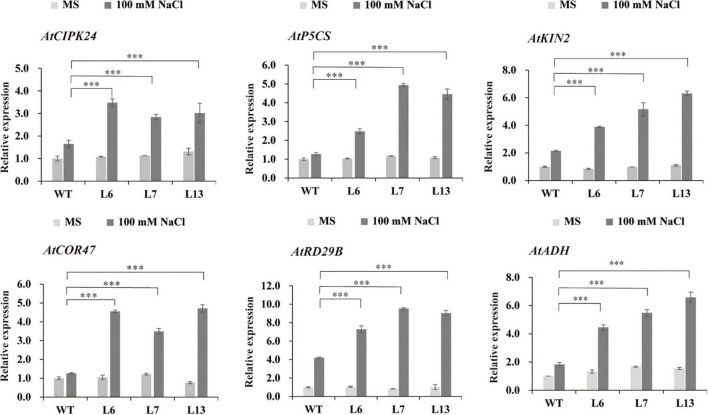
Expression profiles of the six salt stress-responsive genes in WT and *HbERF6*-overexpressing seedlings before and after salt stress using qRT-PCR. The seedlings were grown on MS agar plates for 11 days, and then transferred to MS medium or containing 100 mM NaCl for 10 days. Gene expression was quantified by RT-qPCR and analyzed using the 2^– ΔΔ*CT*^ method. *AtACTIN1* was used as an inter-reference gene. The experiments were repeated three times. Asterisks show that the difference in the values is significant between the transgenic lines and WT at the same time point (^***^P < 0.001).

To know what stress-responsive genes were affected by the overexpression of *HbERF6* in *Arabidopsis*, the expressions of some target genes in whole seedlings were detected using quantitative reverse transcription PCR (qRT-PCR). We analyzed the expression of 12 stress-related genes of interest in *HbERF6*-overexpressing and WT seedlings. According to the qRT-PCR results, the expression levels of *AtCIPK24*, *AtP5CS*, *AtADH*, *AtKIN2*, *AtCOR47*, and *AtRD29B* were upregulated in both of the transgenic and WT seedlings under 100 mM NaCl stress; however, the increase of mRNA abundance of these genes in the transgenic lines was significantly higher than that in WT. Obviously, the expression levels of these genes were not significantly different between transgenic and WT seedlings grown under normal condition (Figure 6). The AP2/ERF transcription factor can bind to one or both of GCC-box (AGCCGCC) and DRE (A/GCCGAC) elements to respond to various biotic or abiotic stresses (Cheng et al., 2013; Lee et al., 2015). Interestingly, among six differentially expressed genes, the promoter of *AtP5CS* contains both GCC-box and DRE elements, and that of *AtCIPK24*, *AtCOR47*, *AtRD29B*, and *AtKIN2* contains DRE elements. However, the promoters of other four genes have neither of them resulting in no differential expression (Supplementary Table 2). These results indicated that the GCC-box or DRE element of the promoter is important for HbERF6 binding and regulation.

## Discussion

### Stress-Responsive Promoter of *HbCIPK2*, a Hub of K^+^/Na^+^ Coordinated Pathways, Was Cloned From Halophytic Wild Barley

Soil salinity substantially impacts plant growth and thus dramatically compromises global agricultural productivity (∼50–80% loss in yield) (Kesten et al., 2019). Discovering novel tolerant genes and unraveling related molecular mechanisms to engineer plants for better stress tolerance are therefore of urgent importance. To date, research on the molecular mechanisms of salt tolerance mainly focuses on the model plants or non-halophytic species. Halophytic grass, especially halophyte without specific structures such as salt-gland, has evolved the mechanism adaptive to salt stress, and attracts more attention (Mansour et al., 2021).

*Hordeum brevisubulatum* is a perennial halophyte relative to cultivated barley, referring to wild barley. Our previous studies indicated that it could maintain higher K^+^ content under salt stress, and HbCIPK2 was further identified to prevent K^+^ reduction and Na^+^ accumulation, and the channel HbVGKC1 and transporter HbSOS1L were coordinated by HbCIPK2 as a hub to maintain K^+^/Na^+^ homeostasis (Li et al., 2012; Zhang et al., 2020a). HbCIPK2 can phosphorylate the targets to activate their activities, which may determine the coordinated signaling pathways to switch on or off. More importantly, *HbCIPK2* is inducible under NaCl and osmotic stress, and which transcriptional factor may modulate HbCIPK2-mediated pathways is a more interesting question. However, genome information for this species is unknown. Therefore, we have to apply the method of genome-walking to clone the *HbCIPK2* promoter, and then confirm that it is a stress-inducible promoter, which is consistent with the expression of *HbCIPK2*, although the signal of the *GUS* gene driven by the *HbCIPK2* promoter is not strong (Figure 1B). This result lays the way for the identification of transcriptional factor for the regulation of HbCIPK2-mediated pathways, especially in halophyte without genome information.

### HbERF6 Was Identified *via* Library Screening Using the *HbCIPK2* Promoter as the Bait

The classical method for the identification of the upstream transcriptional regulator is screening Y1H library using the known promoter sequence as the bait. We found that there were several *cis*-acting elements such as ABRE, CRT/DRE, and MYB in the sequence of *HbCIPK2* promoter, but it is not sure which *cis*-element functions (Figure 1A). Therefore, the full-length *HbCIPK2* promoter was used as the bait to screen the cDNA library of wild barley. Fortunately, HbERF6 was identified as one of the possible regulators with the strong interaction of the *HbCIPK2* promoter. Subsequently, we performed the assays (EMSA, pairwise Y1H, and dual LUC) to confirm that HbERF6 can specifically bind to the GCC-box of the *HbCIPK2* promoter *in vitro* and *in vivo* (Figures 2, 3). In addition, subcellular localization experiments clearly indicated that the HbERF6 protein localized at the nucleus of the plant cell, suggesting that it may play critical roles in transcriptional regulation (Figure 2B). On the other hand, *HbERF6* was also induced by salt, drought, and ABA treatment in wild barley, and the *HbERF6* promoter is also inducible (Figure 4A). These results further show that HbERF6 may be a transcriptional regulator of *HbCIPK2*, the hub of K^+^/Na^+^ homeostasis pathways, in halophytic wild barley.

### HbERF6 Regulates HbCIPK2-Mediated Pathways of K^+^/Na^+^ Homeostasis

The plant-specific AP2/ERF is a large gene family, named after the AP2/ERF domain composed of 60–70 amino acids. Numerous AP2/ERF genes were successfully identified and investigated in plants and have been reported to serve as important regulators in many biological and physiological processes, such as plant morphogenesis, responsive mechanisms to various stresses, hormone signal transduction, and metabolite regulation (Rong et al., 2014; Xie et al., 2019; Girón-Ramírez et al., 2021), and the stress-related downstream genes were regulated by ERFs through direct or indirect binding to the target promoters. However, less is known if the ERF-like transcriptional factor can bind and directly modulate CIPK-like kinase encoding genes. If so, the combination of ERF transcriptional regulation with CIPK signaling pathways will highlight stress-responsive transcription factor networks.

In this study, we reported that HbERF6 is a member of the AP2/ERF family which could directly bind to the GCC-box of the *HbCIPK2* promoter, in order to investigate the role of *HbERF6*. It was overexpressed in *Arabidopsis* wild type due to the difficulty of wild barley genetic transformation. The transgenic lines grew well and exhibited longer roots under salt stress (Figure 5A). More surprisingly, *HbERF6* overexpressing lines can maintain higher K^+^ and reduced Na^+^ content (Figures 5F,G), which is just speculated. Then, the expression of stress-related genes in the WT and transgenic seedlings under salt stress was checked. Interestingly, among five differentially expressed genes, their promoters contain both or either of GCC-box and DRE elements, which indicates that the GCC-box or DRE element of the promoter is important for HbERF6 binding and regulation. It is worth noting that *AtCIPK24* (*SOS2*) accumulated more in *HbERF6* overexpressing lines, which is a classical salt-tolerant gene controlling Na^+^ homeostasis *via* the SOS pathway (Qiu et al., 2002), and its promoter has two DRE elements (A/GCCGAC), which may be so suitable for HbERF6 to bind and activate its higher expression after which K^+^/Na^+^ homeostasis is eventually promoted contributing to salt tolerance in *Arabidopsis* transgenic plants. Although AtCIPK2 is a homolog of HbCIPK2, the expression of *AtCIPK2* was not as high as that of *AtCIPK24* in HbERF6-overexpressing *Arabidopsis* plants. The possible reasons are that the surrounding sequence of the DRE element in the *AtCIPK2* promoter may affect the binding of HbERF6 and the number of DRE elements in the *AtCIPK2* promoter is less than that in *AtCIPK24* (Supplementary Table 2), which needs further investigation.

**FIGURE 7:**
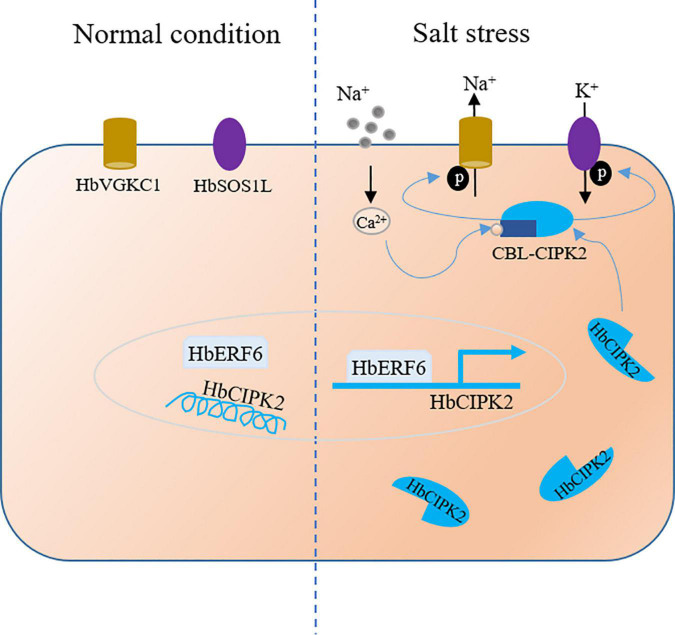
The working model for HbERF6 regulating HbCIPK2-mediated pathways under salt stress. The expression of *HbERF6* is induced by salt conditions. *HbERF6* acts as a transcription factor, binds to the promoter of *HbCIPK2*, and activates the transcription of *HbCIPK2* under salt stress. Then, HbCIPK2 can phosphorylate K^+^ channel HbVGKC1 and Na^+^ transporter HbSOS1L to coordinate K^+^/Na^+^ homeostasis.

Above all, we identified the ERF-mediated regulatory network that integrated transcriptional regulation and post-translation modification, which is a novel salt stress-responsive mechanism for halophytic wild barley. Based on our previous studies and results obtained in this study, the working model of HbERF6 regulating *HbCIPK2* is outlined (Figure 7). In detail, under normal condition, HbERF6 might bind to the *HbCIPK2* promoter and trans-activate *HbCIPK2*, but salt stress significantly induces more HbERF6 protein to bind to the GCC-box of the *HbCIPK2* promoter to enhance its transcription, then HbCIPK2 interacts with the Ca^2+^-sensor HbCBL4/10 to phosphorylate the K^+^ channel HbVGKC1 and Na^+^ transporter HbSOS1L, and finally K^+^/Na^+^ homeostasis in the cell is maintained.

Soil salinity is becoming more and more serious as population increases and climate changes; it is threatening agricultural production and food security. It will be promising to discover available halophytic resources such as wild barley and to improve crop salt tolerance using important tolerant genes such as the HbERF6-HbCIPK2 signaling pathway.

## Data Availability Statement

The original contributions presented in this study are included in the article/Supplementary Material, further inquiries can be directed to the corresponding author.

## Author Contributions

RL, YJ, and HZ planned and designed the experiments. YJ, YL, CC, YW, and HF performed the experiments. YJ wrote the manuscript. RL and HZ revised the manuscript. All authors contributed to the article and approved the submitted version.

## Conflict of Interest

The authors declare that the research was conducted in the absence of any commercial or financial relationships that could be construed as a potential conflict of interest.

## Publisher’s Note

All claims expressed in this article are solely those of the authors and do not necessarily represent those of their affiliated organizations, or those of the publisher, the editors and the reviewers. Any product that may be evaluated in this article, or claim that may be made by its manufacturer, is not guaranteed or endorsed by the publisher.
