# Supplementary material for: A Novel Transcriptional Regulator HbERF6 Regulates the HbCIPK2-Coordinated Pathway Conferring Salt Tolerance in Halophytic Hordeum brevisubulatum
Source: Front Plant Sci. 2022 Jul 7;13:927253. doi: 10.3389/fpls.2022.927253 (PMC9302439; doi:10.3389/fpls.2022.927253)
Supplement: Supplementary file 1 [file Data_Sheet_1.docx]

Supplementary Material


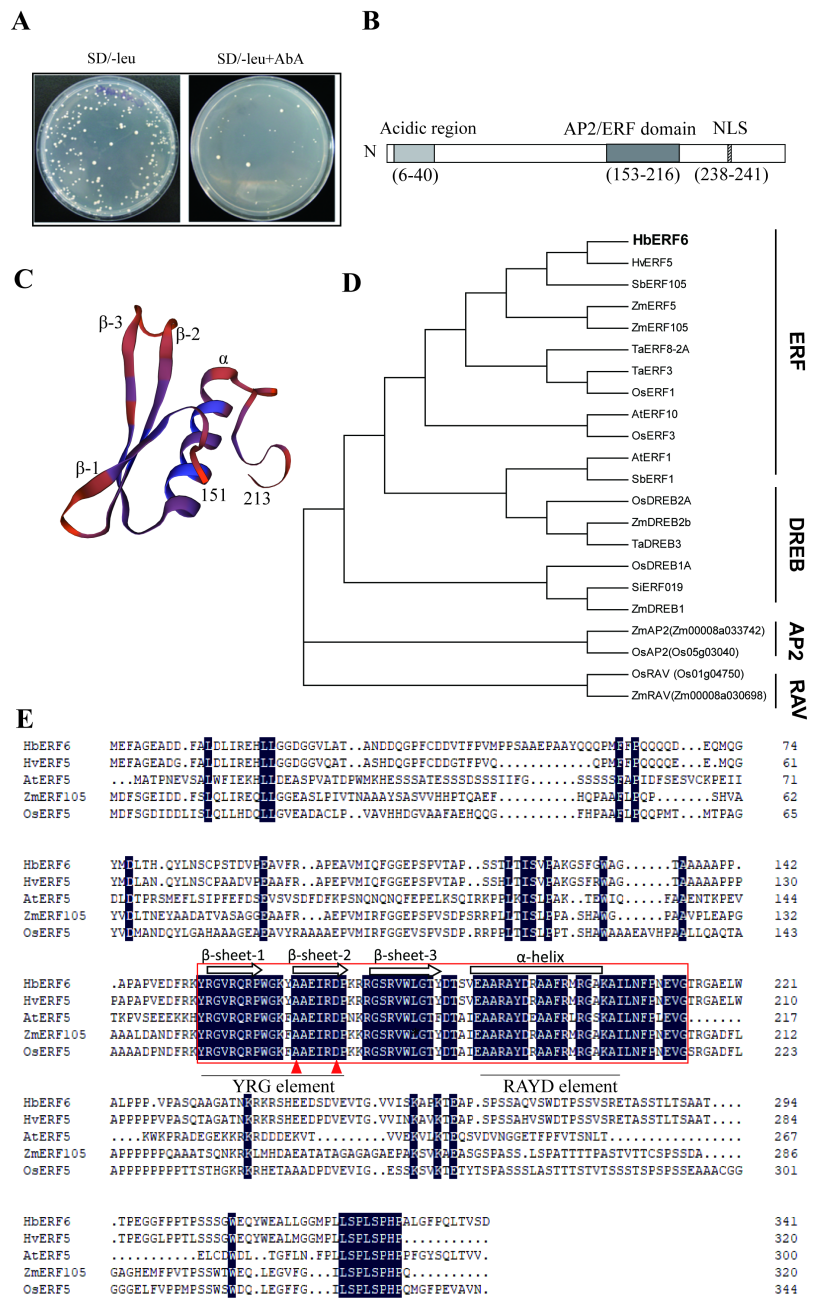
Supplementary Figure 1 Y1H-based library screening with the *HbCIPK2* promoter as the bait and *HbERF6* is identified to one member of the ERF family. (A) The growth situation on SD/-leu and SD/-Leu/AbA（300 mg/L）of transformed yeast with pAbAi-pHbCIPK2. (B) Structural diagram of HbERF6, HbERF6 contains an AP2/ERF domain. (C) Predicted teriary structure of HbERF6 AP2/ERF dormain. (D) Phylogenetic tree of AP2/ERF family proteins. The neighbor-joining method of MEGA 7.0 software was used to analyze the phylogenetic relationship. HbERF6 was marked in bold. (E) Multiple protein sequence alignment of HbERF6 and homologs. The conserved AP2 domains of ERF proteins were marked in red rectangle; both the 14th (A) and the 19th (D) amino acid residues in this domain were indicated by red triangle.


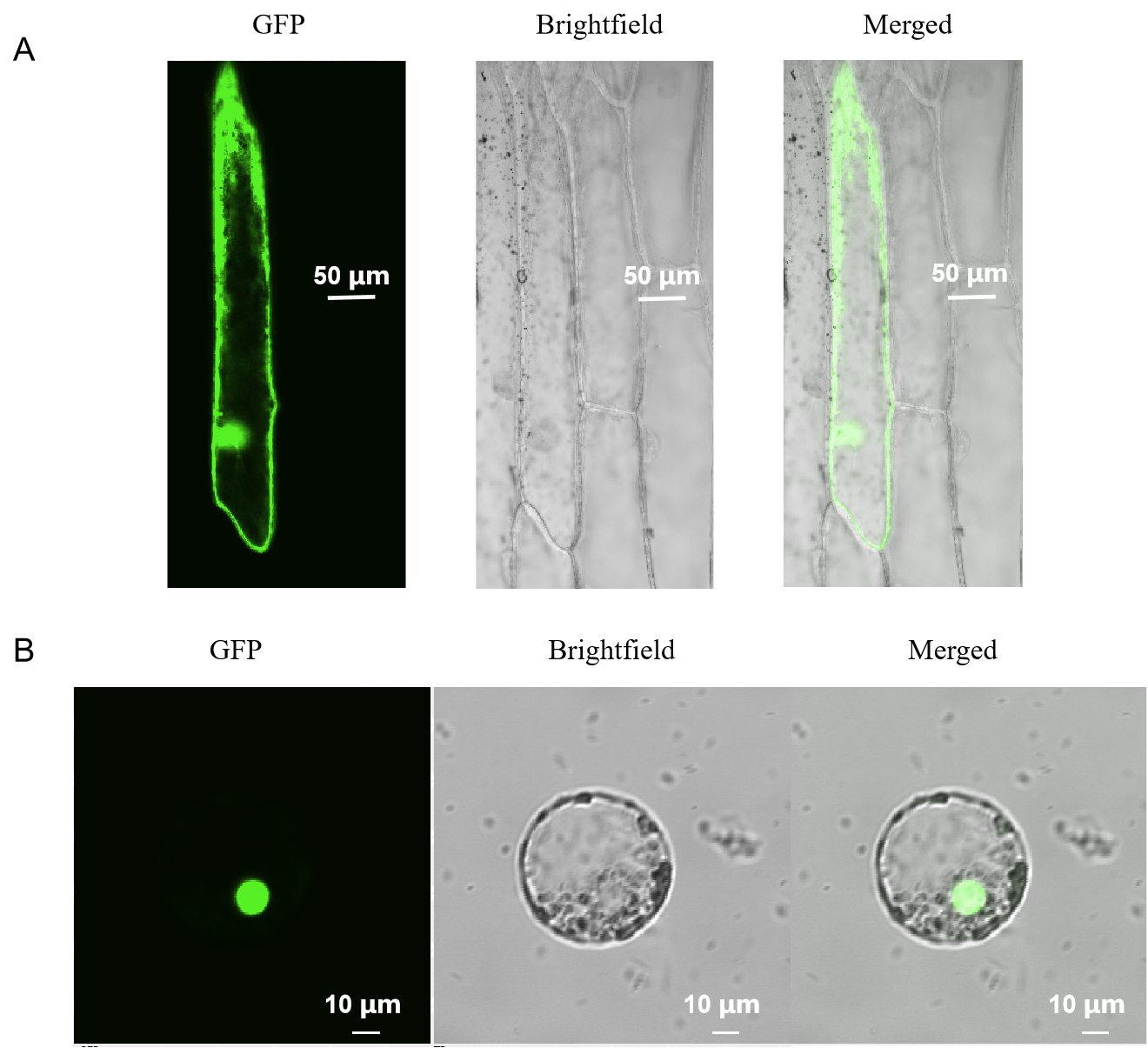


**Supplementary Figure 2** Subcellular localization of GFP and HbERF6-GFP protein in onion epidermal cells and barley (*Hordeum vulagare* L.) protoplasts, respectively. (A) CaMV35::GFP protein is a negative control in onion cells. Bars=50 μm. (B) HbERF6-GFP protein locates in the nucleus of barley protoplasts. Bars=10 μm.

**Supplementary Table 1** Partial cis-acting elements of *HbCIPK2* promoter

| Number | Cis-acting Elements | Position | Core Sequence | Function |
| --- | --- | --- | --- | --- |
| 1 | MYB2CONSENSUSAT | 24 （-）454（+） | YAACKG | Dehydration response element |
| 2 | MYBCORE | 454 （-）962 (+)  1048 (+) 1686 (+) | CNGTTR | Water-stress response element |
| 3 | AGCBOXNPGLB | 673 (+) 1523 (+） | AGCCGCC | CRT/DRE element |
| 4 | DPBFCOREDCDC3 | 718 (+) 988 (-) 1405 （-）1573 （-） | ACACNNG | ABA response element |
| 5 | GT1GMSCAM4 | 732（+）1118（-） | GAAAAA | Salt response element |
| 6 | INRNTPSADB | 795 (+) 1121 (+) 1160 （+） | YTCANTYY | Light response element |
| 7 | PREATPRODH | 834 (+) | ACTCAT | Osmotic pressure response element |
| 8 | MYCATERD1 | 988（+）1614（+） | CATGTG | Dehydration response element |
| 9 | PREATPRODH | 1138 (-) | MACGYGB | Salt response element |
| 10 | MYCCONSENSUSAT | 1238 (-/+)  1301 (-/+) 1356 (-/+) 1614 (-/+) 1757 (-/+) | CANNTG | Dehydration response element |
| 11 | WBOXNTCHN48 | 1271 (+) 1561 (+) | CTGACY | Defense response element |
| 12 | WBOXNTERF3 | 1272 (+) 1562 (+) | TGACY | Wounding response element |
| 14 | CATATGGMSAUR | 1301 (-/+) | CATATG | Auxin  response element |

**Supplementary Table 2** The number and position of GCC-boxes and DRE elements in the promoters of *Arabidopsis* stress-responsive genes

| Genes | Promoter Length (bp)* | GCC-boxes | | DRE elements | |
| --- | --- | --- | --- | --- | --- |
|  |  | Number | Positions from ATG | Number | Positions from ATG |
| *AtCIPK24* | 3000 | 0 | - | 2 | -1151 to -1146, -1080 to -1075 |
| *AtP5CS* | 2811 | 1 | -1209 to -1204 | 1 | -348 to -343 |
| *AtKIN2* | 2392 | 0 | - | 1 | -1462 to -1457 |
| *AtCOR47* | 2570 | 0 | - | 2 | -1103 to -1098, -269 to 264 |
| *AtRD29B* | 650 | 0 | - | 1 | -320 to -315 |
| *AtADH* | 1100 | 0 | - | 0 | - |
| *AtCIPK2* | 2239 | 0 | - | 1 | -549 to -544 |
| *AtLEA3* | 840 | 0 | - | 1 | -666 to -661 |
| *AtPOD* | 1843 | 0 | - | 0 | - |
| *AtNHX1* | 3000 | 0 | - | 0 | - |
| *AtCAT1* | 618 | 0 | - | 0 | - |
| *AtCBL4* | 3000 | 0 | - | 0 | - |

*The promoter sequence of stress-responsive genes in *Arabidopsis* is from ATG start codon to upstream gene terminator codon and the length of promoters is not more than 3000 bp.

**Supplementary Table 3** All the sequence of primers used

| Primer name | Primer sequence 5’-->3’ |
| --- | --- |
| AP1  AP2  SP1  SP2  HbERF6-F  HbERF6-R  HbERF6-GFP-N-F  HbERF6-GFP-N-R  T7-F  3AD-R  30a-ERF6-E-F  30a-ERF6-S-R  CK2pro-1-B-F  CK2pro-1-B-R  CK2pro-2-B-F  CK2pro-2-B-R  CK2pro-1-M-F  CK2pro-1-M-R  CK2pro-1-F  CK2pro-1-R  CK2pro-2-F  CK2pro-2-R  CK2pro-1-M-F  CK2pro-1-M-R  CIPK2pro-LUC-SalI-F  CIPK2pro-LUC-NcoI-R  AtActin1-F  AtActin1-R  AtSOD-F  AtSOD-R  AtCAT1-F  AtCAT1-R  AtLEA-F  AtLEA-R  AtP5CS-F  AtP5CS-R  AtNHX1-F  AtNHX1-R  AtACT2-F  AtACT2-R  AtRD29B-F  AtRD29B-R  AtKIN2-F  AtKIN2-R  AtCOR15a-F  AtCOR15a-R  AtADH-F  AtADH-R  AtFRY1-F  AtFRY1-R  AtCIPK24-F  AtCIPK24-R  AtCBL4-F  AtCBL4-R  AtCIPK2-F  AtCIPK2-R  CIPK2pro-SalI-F  CIPK2pro-NcoI-R  ERF6-SalI-F  ERF6-XbaI-R  HbERF6pro-1381-F  HbERF6pro-1381-R  42AD-ERF6-F  42AD-ERF6-R  Lac-CK2pro-1-F  Lac-CK2pro-1-R  Lac-CK2pro-2-F  Lac-CK2pro-2-R  Hb-qRT-ERF6-F  Hb-qRT-ERF6-R  18SrRNA-3-F  18SrRNA-3-R | GTAATACGACTCACTATAGGGC  ACTATAGGGCACGAGTGGT  CGACGCTCTGCGAGGTCTCGATGTTGCGG  CCGAGCATCTTCCCCATCTCGTACTTGTGC  ATGGAGTTCGCCGGAGAAGCCGAC  AGTCACGGTGAGCTGCGGGAACCC  GTCGACATGAGTAAAGGAGAAG  AAGCTTTTATTTGTATAGTTCATCC  TAATACGACTCACTATAGGG  AGATGGTGCACGATGCACAG  GAATTCATGGAGTTCGCCG  GTCGACTTAAGTCACGGTGAGC  AGGGTAGCCGCCCCAGAGGGTAGCCGCCCCAGAGGGTAGCCGCCCCAG  CTGGGGCGGCTACCCTCTGGGGCGGCTACCCTCTGGGGCGGCTACCCT  AACGGAGCCGCCTCTCAACGGAGCCGCCTCTCAACGGAGCCGCCTCTC  GAGAGGCGGCTCCGTTGAGAGGCGGCTCCGTTGAGAGGCGGCTCCGTT  AGGGTAACCACTCCAGAGGGTAACCACTCCAGAGGGTAACCACTCCAG  CTGGAGTGGTTACCCTCTGGAGTGGTTACCCTCTGGAGTGGTTACCCT  AGGGTAGCCGCCCCAGAGGGTAGCCGCCCCAGAGGGTAGCCGCCCCAG  CTGGGGCGGCTACCCTCTGGGGCGGCTACCCTCTGGGGCGGCTACCCT  AACGGAGCCGCCTCTCAACGGAGCCGCCTCTCAACGGAGCCGCCTCTC  GAGAGGCGGCTCCGTTGAGAGGCGGCTCCGTTGAGAGGCGGCTCCGTT  AACGGAACCACTTCTCAACGGAACCACTTCTCAACGGAACCACTTCTC  GAGAAGTGGTTCCGTTGAGAAGTGGTTCCGTTGAGAAGTGGTTCCGTT  GTCGACGCCAGTGCTACTACTACTG  CCATGGGGTGGCAGGCAGAT  GGCGATGAAGCTCAATCCAAACG  GGTCACGACCAGCAAGATCAAGACG  CGCATGATCCTTTGGCTTCG  TCCTGGTTGGCTGTGGTTTC  TCCTGTTATCGTTCGTTTCTCA  CAAAGTTCCCCTCTCTGGTGTA  GATTGACCCGGCTGAGCTACGA  AGATGGGATTCACCACAAAAGA  GGGACAAGTTGTGGATGGAGAC  TGGTACAAACCTCAAGGAACAC  AGCCTTCAGGGAACCACAAT  CTCCAAAGACGGGTCGCATG  TCGCTGACCGTATGAGCAAAG  TGTGAACGATTCCTGGACCTG  GTGAAGATGACTATCTCGGTGGTC  TACCAAGAGACTCAGCAATCTCTG  GTCAGAGACCAACAAGAATGCC  TGACTCGAATCGCTACTTGTTC  ACTCAGTTCGTCGTCGTTTCTC  TCTCACCATCTGCTAATGCCTC  CTCTTGGTGCTGTTGGTTTAGG  AATTGGCTTGTCATGGTCTTTC  CGCAGTAGCACTAGGATTG  TTGACACCGAGTTTATTGG  ATTGAGGCTGTAGCGAAC  GGTATTCCTTCTGTTGCC  GGAGGAATCTCTTCGCTG  CACGAAAGCCTTATCCACC  TAAGTGCGCTTGCTGATTGC  CCGCTTTCGTACCCTCGTAT  GTCGACGCCAGTGCTACTACTACTG  CCATGGGGTGGCAGGCAGAT  GTCGACATGGAGTTCGCCGGA  TCTAGATTAAGTCACGGTGAGCTGC  tgggcccggcgcgccgaattcGTTGCGCCGGAATCGGTC  ggtggactcctcttaaagcttGCCGCCTAAGAGGGATCG  gattatgcctctcccgaattcATGGAGTTCGCCGGAGAAGC  agaagtccaaagcttctcgagTTGATCACTCACGGTGAGCTGC  AGGGTAGCCGCCCCAGAGGGTAGCCGCCCCAGAGGGTAGCCGCCCCAG  TCGACTGGGGCGGCTACCCTCTGGGGCGGCTACCCTCTGGGGCGGCTACCCTGTAC  AACGGAGCCGCCTCTCAACGGAGCCGCCTCTCAACGGAGCCGCCTCTC  TCGAGAGAGGCGGCTCCGTTGAGAGGCGGCTCCGTTGAGAGGCGGCTCCGTTGTAC  CGGAGAAGCCGACGACTT  GAAGGTGGCATCACAGGG  TTTCGTGAGGGCCTGCTTAG  GACTCACAGAACATGGGGCA |
